# Molecularly Imprinted Nanoparticles Assay (MINA) in Pseudo ELISA: An Alternative to Detect and Quantify Octopamine in Water and Human Urine Samples

**DOI:** 10.3390/polym11091497

**Published:** 2019-09-13

**Authors:** Ewa Moczko, Richard Díaz, Bernabé Rivas, Camilo García, Eduardo Pereira, Sergey Piletsky, César Cáceres

**Affiliations:** 1 Departamento de Química Ambiental, Facultad de Ciencias, Universidad Católica de la Santísima Concepción, Concepción 4090541, Chile; ewa@ucsc.cl; 2 Departamento de Polímeros, Facultad de Ciencias Químicas, Universidad de Concepción, Concepción 4070371, Chile; richdiaz@udec.cl (R.D.); brivas@udec.cl (B.R.); 3 Departamento de Ciencias Biológicas y Químicas, Facultad de Recursos Naturales, Universidad Católica de Temuco, Temuco 4781312, Chile; cgarcia@uct.cl; 4 Departamento de Química Analítica e Inorgánica, Facultad de Ciencias Químicas, Universidad de Concepción, Concepción 4070371, Chile; epereira@udec.cl; 5 Chemistry Department, College of Science and Engineering, University of Leicester, Leicester LE1 7RH, UK; sp523@leicester.ac.uk

**Keywords:** molecularly imprinted nanoparticles assay, molecularly imprinted polymers, ELISA, doping, octopamine

## Abstract

In 2004, octopamine was added to the list of drugs banned by the world anti-doping agency (WADA) and prohibited in any sport competition. This work aims to develop a new analytical method to detect octopamine in water and human urine samples. We proposed a pseudo-enzyme-linked immunosorbent assay (pseudo-ELISA) by replacing traditional monoclonal antibodies with molecularly imprinted polymer nanoparticles (nanoMIPs). NanoMIPs were synthesised by a solid-phase approach using a persulfate initiated polymerisation in water. Their performance was analysed in pseudo competitive ELISA based on the competition between free octopamine and octopamine-HRP conjugated. The final assay was able to detect octopamine in water within the range 1 nmol·L^−1^–0.1 mol·L^−1^ with a detection limit of 0.047 ± 0.00231 µg·mL^−1^ and in human urine samples within the range 1 nmol·L^−1^–0.0001 mol·L^−1^ with a detection limit of 0.059 ± 0.00281 µg·mL^−1^. In all experiments, nanoMIPs presented high affinity to the target molecules and almost no cross-reactivity with analogues of octopamine such as pseudophedrine or l-Tyrosine. Only slight interference was observed from the human urine matrix. The high affinity and specificity of nanoMIPs and no need to maintain a cold chain logistics makes the nanoMIPs a competitive alternative to antibodies. Furthermore, this work is the first attempt to use nanoMIPs in pseudo-ELISA assays to detect octopamine.

## 1. Introduction

The essential and conventional methods to detect and analyse biomolecules are the immunoassays. ELISA is one of the most common immunoassay for a wide range of diagnostics. It is based on a competition between free and labelled ligand to bind to immobilised receptors, where the analytical signal usually depends on a colourimetric or fluorescent reaction [1]. Additionally, advantageous for ELISA assays is that they can be performed for a vast number of analytes. Although this technique has revealed great selectivity and sensitivity, ELISA still suffers from tedious assay procedures and low stability. This is mainly due to changes in environmental conditions, which affects antibodies that are easy denaturised with changes of pH, ionic strength or temperature. Another major issue related to the use of ELISA is the high cost of the assay (mainly due to the high price of the antibodies or the antigens [2]. Therefore, there is still a need to find new materials that can solve the problems of using antibodies or antigens, and at the same time maintain highly selective molecular recognition.

Over the last few years, the idea of replacing antibodies with artificial receptors (molecularly imprinted polymers, MIPs) has become physically possible due to advancement in solid-phase synthesis of MIP nanoparticles [3,4,5,6,7]. MIPs are a category of synthetic receptors, where co-monomers are polymerised in the presence of a target analyte, so-called template molecule. The template is subsequently removed from the polymeric matrix, leaving a cavity, which is geometrically and chemically complementary to the template. In this way, they are able to specifically rebind the targets [8,9]. Additionally, when MIPs are compared with the natural receptors, they show similar affinity to the analytes and highly improved physicochemical properties. Therefore, MIPs do not need a cold chain logistics and also they can be easily integrated with electronics [10,11,12,13]. Due to these reasons, the replacement of antibodies with MIPs in ELISA assay was considered for the first time in the late 1990s [14]. The method of integration of MIPs in the ELISA microplate was accomplished by straight polymerisation of the MIP on the bottom of the microplate or by grafting the previously synthesised polymer on the base of the well [15,16,17,18,19]. Despite easy implementation, this method of immobilisation suffered from the lack of reproducibility. Recently advances in MIP synthesis, such as precipitation polymerisation [20] or solid-phase synthesis [21], are solving those problems by scaling down the material to the nanometer scale (20–250 nm) [22,23,24,25]. NanoMIPs has attracted widespread interest as they can be used for the production of robust and remarkably selective synthetic receptors for a diversity of molecules ranging from proteins to small organic molecules [26,27,28]. Recently nanoMIPs were used as “plastic antibodies” to replace traditional monoclonal antibodies in ELISA. The idea was already proven in the sensing of vancomycin in pork fluids in 2013 [29]. Nowadays, there are several examples of the successful use of nanoMIPs to replace antibodies in ELISA for detection of small organic molecules or large proteins [26,30]. The limits of detection were always comparable or better to these of standard ELISA [31]. In this work, we are demonstrating the performance of nanoMIPs in a pseudo ELISA assay (MINA) as a precursor for the detection of prohibited substances in sport.

NanoMIPs were synthesised in water using the approach of solid-phase with an immobilised template. After characterisation, they were applied in the pseudo-ELISA assay (MINA) to detect octopamine. Octopamine is a biogenic amine [4-(2-amino-1-hydroxyethyl)phenol] that normally occurs in plants, mollusks and vertebrates [32]. It was firstly isolated from the salivary organs of *Octopus vulgaris* [33]. Octopamine can be found in the serum of humans in measurable (µg·mL^−1^) amount with certain alterations in the case of innard and neurological diseases [34]. In 2004 octopamine was classified as a stimulant and listed by the world anti-doping agency (WADA) as a prohibited substance for all athletes in sports competitions [35]. It was due to its strong effect in mammals causing stimulation of the central nervous system, enhancement of the anti-inflammatory effects of corticosteroids and general performance. Additionally, similar to other biogenic amines, octopamine is efficient partitioning agents that promote body fat reduction and enhance animal growth [36]. Normally the doping analysis of octopamine in human urine has been based on a sample arrangement applying cation exchange solid-phase extraction (SPE-XCW) accompanied by liquid chromatography–tandem mass spectrometry (LC–MS/MS) [37]. Using this advanced technique, it was possible to obtained LOD 0.46 µg·mL^−1^ [38]. There are also available commercial kits for the detection of octopamine in humans, which use conventional ELISA [39]. The major drawback of those types of analysis is the high cost and the limitations of working with biological samples. Therefore, it is important to search for new solutions and try to develop new assays, such as MINA for easier and cost-effective detection.

## 2. Materials and Methods

### 2.1. Materials

Acrilic Acid (AA), ammonium persulfate (APS), bovine serum albumin (BSA), 1-ethyl-3-(3-dimethylaminopropyl)-carbodiimide hydrochloride (EDC), glutaraldehyde (GA), 3-aminopropyltrimethyloxysilane (APTMS), horseradish peroxidase (HRP), sodium hydroxide (NaOH), sodium cyanoborohydride, *N*-hydroxysuccinimide (NHS), phosphate buffered saline (PBS), 3,3′,5,5′-tetramethylbenzidine (TMB), *N*,*N*′-methylene-bis-acrylamide (BIS), *N*-isopropylacrylamide (NIPAm), *N*-tert-butylacrylamide (TBAm), tetramethyl ethylene diamine (TEMED), octopamine hydrochloride, Tween 20, 2-[morpholino] ethanesulfonic acid (MES), and [3-(2-aminoethyl- amino)propyl] trimethoxysilane were purchased from Sigma-Aldrich (Concepción, Chile).

Double-distilled ultra-pure water (Milli-Q, Concepción, Chile) was used in all the experiments. Acetone, ethanol, methanol and toluene were purchased by MERCK. All chemicals and solvents were of analytical or HPLC grade and were used without further purification. Microplates used were Nunclon 96 microwell plates (Thermo Scientific, Concepción, Chile), Amicon centrifugal filter unit (MWCO 30 kDa, Chile).

### 2.2. Preparation of Solid-Phase for Octopamine

The procedure for the conditioning of the solid-phase for immobilisation of compounds with a primary amino group has already been described before [29,40]. Briefly, glass beads were activated by boiling with 1 mol·L^−1^ sodium hydroxide for 15 min, washed with Milli-Q water until the pH of the supernatant was between 6.0–7.0. Afterwards, the glass beads were rinsed with acetone and dried at 60 °C for 4 hours. Later they were incubated in a 2% *v/v* solution of APTMS in anhydride toluene at ambient temperature for 24 hours. Afterwards, the glass beads were filtered, flushed with acetone and methanol, and finally dried. At this point, the modified glass beads are stable for 6 months.

The surface attachment of octopamine on the glass beads was obtained by chemical reaction between the free primary amine of the silane group on the glass beads and the carbonyl of GA. The silanized glass beads were incubated for 2 hours in a solution of GA (7% *v/v*) in PBS pH 7.2. Then the glass beads were filtered, washed with Milli-Q water and incubated in 5 mg·mL^−1^ of octopmine in 0.01 mol·L^−1^ PBS (pH 7.4) overnight at ambient temperature. The reductive alkylation was performed for 30 min by incubating glass beads in 1 mg·mL^−1^ solution of sodium cyanoborohydride in 0.01 mol·L^−1^ PBS at ambient temperature. This method yields 0.26 ligand molecules per nm^2^ of glass beads surface [21]. Finally, the glass beads with immobilized octopamine were washed with Milli-Q water, dried under vacuum and stored at 4 °C until used.

### 2.3. Synthesis and Purification of NanoMIPs-O

The synthesis of nanoMIPs was performed by mixing: 39 mg of NIPAM (0.34 mmol), 2 mg of BIS (0.013 mmol), 33 mg of TBAm, dissolved in 1 mL of ethanol (0.26 mmol), and 2.2 µL of AA (0.032 mmol). The components were dissolved in Milli-Q water (100 mL). The solution was sonicated for 15 min and degassed by bubbling with nitrogen for 30 min. Subsequently, 60 g of glass beads functionalized with octopamine were added to the glass reactor which contains 50 mL of polymerisation mixture and briefly mixed to homogenise the contents. The mixture was again degassed with nitrogen for 25 min. The radical polymerisation reaction was started by adding 600 μL of APS (60 mg·mL^−1^) and 18 μL TEMED and left to react for 1.5 h at ambient temperature. During polimerisation, gentle stirring was applied at the end of each 30 min. Subsequently, the polymerisation mixture was removed from the reaction vessel using suction and nitrogen purging. Finally, the unreacted monomers, small oligomers and low-affinity materials were removed by washing with cold water at 4 °C (60 mL × 8 times). The high-affinity nanoMIPs were collected using Milli-Q water (60 mL × 5 times) at 60 °C. The solutions of high-affinity nanoMIPs were concentrated till 100 mL by ultrafiltration on a Millipore Amicon Ultra centrifugal filter unit (30 kDa MWCO) and used in the pseudo-ELISA MINA assay.

### 2.4. Characterisation of NanoMIPs

The concentration of the nanoMIPs solution was determined based on a calibration curve obtained by evaporating different aliquots of the nanoMIP solution and weighing dry sample. The size of the nanoMIPs was determined by using a dynamic light scattering analyser (DLS) from Brookhaven Instruments Corporation Ltd. (Holtsville, NY, USA) and images obtained on a transmission electron microscope (TEM) from JEOL/JEM 1200 EX II (Tokyo, Japan). Before DLS and TEM analysis, the solution of nanoMIPs was sonicated 10 min, and measurements were performed at 25 °C. DLS was performed in 1 ml of the solution of nanoMIPs in water using 3 cm^3^ disposable polystyrene cuvette. For TEM measurements, 20 μL of the nanoMIPs dispersion was placed on a carbon-coated copper grid and dried in ambient temperature under a fume hood.

### 2.5. Devolpment of MINA

#### 2.5.1. Preparation of HRP-Octopamine (HRP-O) Conjugate

The protocol for the synthesis of the conjugate has been described before [26]. Briefly, octopamine was activated using a stock solution at the concentration of 0.2 mg·mL^−1^ in 0.1 M MES buffer pH 6. Then 17.7 μL of EDC dissolved in water were taken from 10 mg·mL^−1^ stock solution and added to the solution, followed by NHS (1.72 mg). The reaction proceeds at ambient temperature for 15 min. Subsequently, the solution was mixed with 20 mL of HRP (0.6 mg·mL^−1^) in PBS buffer at pH 7.4 and incubated for 2 h. After that, the HRP-O conjugate was carefully washed with water using a Millipore Amicon Ultra centrifugal filter unit (30 kDa MWCO) in order to remove all free octopamine. In this procedure, ten washes with PBS (5 mL) were performed. After washing, the conjugate was suspended in 2 ml of Milli-Q water. The concentration of the conjugate was estimated by comparison with the enzymatic activity of the free enzyme. Finally, the conjugate was stored at −18 °C and used as the stock solution in the pseudo-ELISA tests. 

#### 2.5.2. Immobilization of NanoMIPs onto the Surface of Microplate Wells

The immobilisation of nanoMIPs onto the microplate wells was performed by direct deposition of the nanoparticles’ solutions (40 μL, 0.056 mg·mL^−1^) into the wells of a 96-well microplate. After dispensing, the solvent was evaporated overnight at ambient temperature.

#### 2.5.3. Optimisation of MINA Conditions

Several parameters, such as the composition of blocking and washing buffers, time of the assay with TMB, and stopping solution were adopted from Chianella et al. [29]. The concentration of HRP-O and the concentration of the nanoMIPs was optimised to improve the response of the assay.

#### 2.5.4. Optimisation of HRP-O Conjugate Concentration

Each microplate well was coated with nanoMIPs by dispensing undiluted stock solution (40 μL, 0.056 mg·mL*^−^*^1^) followed by overnight evaporation. Each well was conditioned by washing with 0.01 mol·L*^−^*^1^ PBS (2 × 250 μL), followed by 1 h blocking with the solution of 300 μL of 0.01 mol·L*^−^*^1^ PBS containing 0.1% of BSA and 1% of Tween 20. After further washings with 0.01 mol·L*^−^*^1^ PBS (3 × 250 μL), 100 μL of HRP-O conjugate was added using several dilutions (1:200, 1:600, 1:800, 1:1000, 1:1200 and 1:1600). The microplates were incubated in the dark at ambient temperature for 1 h. After washing with 0.01 mol·L^−1^ PBS (3 × 300 μL), containing 0.1% of BSA and 1% of Tween 20, TMB reagent (100 μL) was added to the wells and incubated for 10 min. The enzymatic reaction was stopped by the addition of H_2_SO_4_ (0.5 mol·L*^−^*^1^, 100 μL). The absorbance of each microplate well was measured at 450 nm using the UV/Vis microplate reader.

#### 2.5.5. Optimisation of NanoMIP Concentration

Each microplate well was coated with nanoMIPs by dispensing 40 μL of different concentrations varying from 0.00056 to 0.56 mg·mL*^−^*^1^ followed by overnight evaporation. Further, each well was conditioned by washing with 0.01 mol·L*^−^*^1^ PBS (2 × 250 μL) followed by 1 h blocking with 300 μL of 0.01 mol·L*^−^*^1^ PBS containing 0.1% of BSA and 1% of Tween 20. After further washings with 0.01 mol·L*^−^*^1^ PBS (3 × 250 μL), 100 uL of HRP-O conjugate (1:1200) was added to each well. The microplates were incubated in the dark at ambient temperature for 1 h. After washings with 0.01 mol·L*^−^*^1^ PBS (3 × 300 μL), containing 0.1% of BSA and 1% of Tween 20, TMB reagent (100 μL) was added and incubated for 10 min. The enzymatic reaction was stopped by the addition of H_2_SO_4_ (0.5 mol·L*^−^*^1^, 100 μL) and the absorbance of each microplate well was measured at 450 nm using the UV/Vis microplate reader (Figure S3).

#### 2.5.6. Competitive MINA for the Determination of Octopamine

The final conditions for MINA assay are described in Table 1. A 96-well microplate was coated with nanoMIPs by dispensing 40 μL of 0.056 mg·mL*^−^*^1^ into each well, followed by overnight evaporation. After that, each well was conditioned by washing with PBS (2 × 250 μL), followed by 2 h blocking with 300 μL of 0.01 mol·L*^−^*^1^ PBS containing 0.1% of BSA and 1% of Tween 20. After further washing with 0.01 mol·L*^−^*^1^ PBS (3 × 250 μL), 100 uL of HRP-O conjugate (1:1200) was mixed with the standard solution of free octopamine from (1 nmol·L*^−^*^1^–0.0001 mol·L^−1^). The microplates were incubated in the dark at ambient temperature for 1 h. After washing with 0.01 mol·L*^−^*^1^ PBS (3 × 300 μL), containing 0.1% of BSA and 1% of Tween 20, TMB reagent (100 μL) was added and incubated for 10 min. The enzymatic reaction was stopped by the addition of H_2_SO_4_ (0.5 mol·L*^−^*^1^, 100 μL) and the absorbance of each microplate well was measured at 450 nm using the UV/Vis microplate reader.

#### 2.5.7. Analysis of Octopamine in Human Urine Samples

We confirm that all methods were carried out in accordance with relevant guidelines and regulations. Furthermore, the human urine was obtained from a healthy volunteer over 18 years old with full consent. All the experimental protocol related to the use of human urine was approved by an institutional committee of Bioethics from the University of Concepcion. This part of the work was performed in order to demonstrate the capability of the new assay, MINA, to detect octopamine in real biological media. Therefore, urine was spiked with octopamine at the concentrations covering the clinical range. The stock solution of urine was diluted (1:1, 1:10, 1:100 and 1:1000) decanted and filtrated with a 0.22 μm syringe filter (PVDF). Octopamine concentration in urine samples was determined using the competitive assay described above (details of the procedure are included in Table 1). To demonstrate the clinical use of the assay, the concentrations of octopamine were determined based on absorbance and the calibration curve of a competition between HRP-O and free octopamine.

#### 2.5.8. Cross-Reactivity of the MINA Assay for Octopamine

In order to evaluate the cross-reactivity and the selectivity of the MINA assay, the competitive assay was performed with three molecules two analogues of octopamine, l-tyrosine and pseudoephedrine, and labetalol, which is another prohibited compound in the sport. The cross-reactivity assay was performed in human urine samples

## 3. Results and Discussion

### 3.1. Synthesis and Characterisation of NanoMIPs

**Figure 1:**
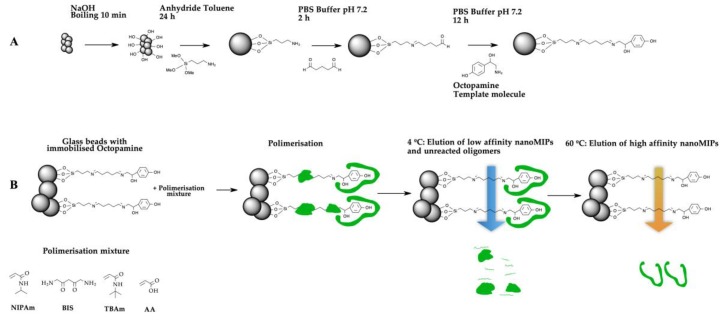
(**A**) Procedure for the immobilisation of octopamine on the surface of the solid support (glass beads). (**B**) Scheme of the synthesis of molecularly imprinted polymer nanoparticles (nanoMIPs) made for octopamine. Low-affinity particles and oligomers of the synthesis were washed at 4 °C. High-affinity nanoMIPs were collected at 60 °C.

In order to obtain cost-effective and robust macromolecular receptors, we performed the synthesis of nanoMIPs for octopamine using a solid phase approach (Figure 1). Firstly, the glass beads were activated by boiling with NaOH. Subsequently, the glass beads were conjugated with APTMS adding to the surface a primary amine. Further, the amino glass beads were derivatised with GA; this reaction is allowed by nucleophilic addition of the amino group to the carbonylic carbon of the GA to produce a stable imine. Finally, octopamine was immobilised on the glass-beads by nucleophilic addition of the amino group of the octopamine to the carbonylic carbon of the glutaraldehyde glass beads [21]; see Methods section. 

After the template molecule was attached to the glass beads, the polymerisation was carried out in aqueous media. The polymer composition for the preparation of the nanoMIPs was adopted from Hoshino et al [41]. In general, the interactions between the polymerisation mixture and the template molecule were due to a combination of a different hydrophobic, electrostatic and hydrogen bond interactions. 

To carry out the synthesis of the nanoMIPs, the polymerisation mixture (NIPAm, BIS, TBAm and AA) was added to the glass beads-Octopamine, and the radical polymerisation reaction was initiated by the addition of the initiation mixture (APS and TEMED). After two hours of reaction, we proceed to wash the nanoMIPs. The optimal washing temperatures of nanoMIPs have been described in previous works [26,29]. Consequently, first washings with Milli-Q were performed at 4 °C to remove particles with less affinity, and traces of nonreacted monomers. Then at 60 °C high-affinity nanoparticles were eluted, collected and stored for a further use. The final concentration of the stock solution of nanoMIPs was determined by weighing a freeze-dried aliquot of the nanoparticles solution and, if needed, it was adjusted to the concentration of 0.056 mg·mL^−1^. 

**Figure 2:**
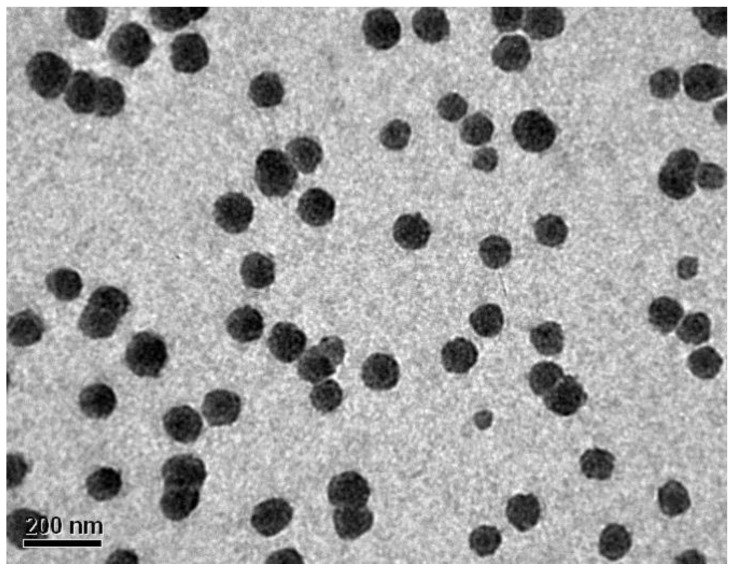
TEM image of nanoMIPs for octopamine.

The size of the nanoparticles, measured by DLS was 137 ± 2.210 nm with a polydispersity of 0.3205 ± 0.0121 (Table S1, Figure S1). The low polydispersity of the nanoMIPs indicates a homogeneous size distribution. The nanosize and the shape of the nanoparticles was confirmed by TEM results (Figure 2).

### 3.2. Development of MINA

After the synthesis and characterisation of the nanoMIPs, they were used as a replacement of antibodies in pseudo-ELISA to develop a quantitative assay for the detection of octopamine in water and human urine samples. 

The immobilisation of the nanoMIPs was performed in a very similar way as the immobilisation of antibodies, through physical absorption onto the walls of polystyrene microplates.

The immobilisation of the quantity of nanoMIPs was optimized (Figure S2) and it was obtained by a simple overnight evaporation that allowed nanoparticles to remain attached to the microplate well surface even after several washes with PBS.

**Figure 3:**
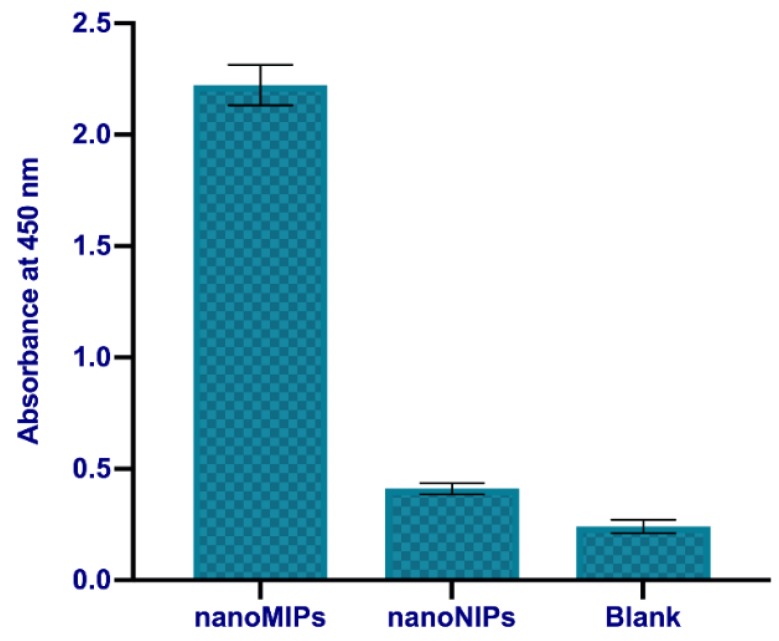
Binding of the horseradish peroxidase-octopamine (HRP-O) conjugate to the nanoMIPs immobilised in a microplate and uncoated microplates. Error bars represent the standard deviation for experiments performed in triplicate.

To test the affinity of immobilised nanoMIP for otopamine, the HRP-O was added to the coated wells and incubated. After that, the solution was washed out and the results were analysed based on the color development during the chemical reaction with TMB. Control experiments were performed using uncoated microplates. Additionally, to test the specificity of nanoMIPs, we compared the binding of HRP-O to nanoNIPs imprinted for labetalol, another banned molecule. The results were reported in Figure 3. It can be seen that much higher binding of HRP-O was observed in the case of the nanoMIPs prepared for octopmine, than either nanoNIPs or bare plates. 

Once immobilised, the nanoMIPs were used in a competitive pseudo-ELISA assay to quantify octopamine through a competition between a different concentration of free octopamine and HRP-O. The assay in water and urine samples were performed using the same conditions as in the assay previously developed for vancomicyn [29]. The first part of the protocol involves the determination of the optimal dilutions of the HRP-O. 40 μL of the solution of nanoMIPs for octopamine (0.056 mg·mL^−1^) were immobilised in each microplate well. After evaporation of the solvent, the optimal dilution of HRP-O was determined at 1:1200 (Figure S2). 

**Figure 4:**
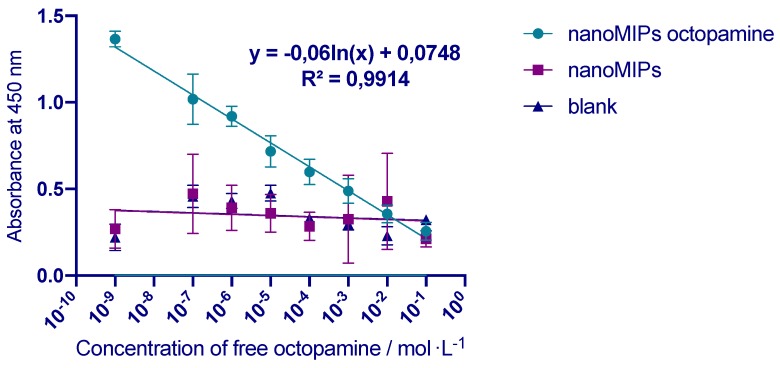
Calibration curve for MINA assay. Light blue line indicates binding of octopamine to octopamine specific nanoMIPs (circles). Purple line indicates binding of octopamine with labetalol specific nanoNIPs (squares). Dark blue line indicates binding of octopamine to blank, uncoated microplates. Error bars represent the standard deviation for experiments performed in triplicate.

Afterwards, the first competitive assay was performed in water. Initially, microplates were coated with nanoMIPs imprinted for octopamine, following the procedures in Table 1. Next, the calibration curve with different concentrations of free octopamine was performed and presented in Figure 4. Each point of the calibration curve was obtained for different concentration of free octopamine using the same concentration of HRP-O (1:1200 dilution). Therefore, the free analyte and the analyte marked with the HRP were competing for the same active site in the nanoparticles. It was demonstrated that absorbance was related to the concentration of free octopamine. The assay prepared for octopamine showed a linear response in the concentration range of 1 nmol·L^−1^–0.1 mol·L^−1^. The results indicated that free octopamine could be detected over 8 orders of magnitude concentration range (r^2^ = 0.9914) when plotted on a logarithmic scale. The limits of the method were calculated by the IUPAC recommendations $LOD/LOQ=\frac{F*SD}{b}$ where F is a factor of 3.3 and 10 for LOD and LOQ respectively, SD is the standard deviation of the blanks and b is the slope of the regression line. The limit of detection (LOD) is 0.047 ± 0.00231 µg·mL^−1^ and it was calculated from 24 times value of the standard deviation of the control (in the absence of octopamine), the limit of quantification was 0.1551 ± 0.00231 0.0076 µg·mL^−1^. The competitive assay showed saturation at the concentration of octopamine higher than 0.1 mol·L^−1^. The assay was repeated during the same day and on different days with a very good repeatability with %RSD inferior to 5%.

The next step was to evaluate the selectivity of the MINA for octopamine. In order to do that, nanoNIPs imprinted for labetalol were immobilised in the microplates and different aliquots of free octopamine and HRP-O were added and measured. The results are presented in Figure 4. It can be seen that in the case of different types of nanoNIPs, no specific binding to octopamine was observed. Similar results were observed using blank, uncoated microplates. Therefore, the results revealed significant selectivity of nanoMIPs.

**Figure 5:**
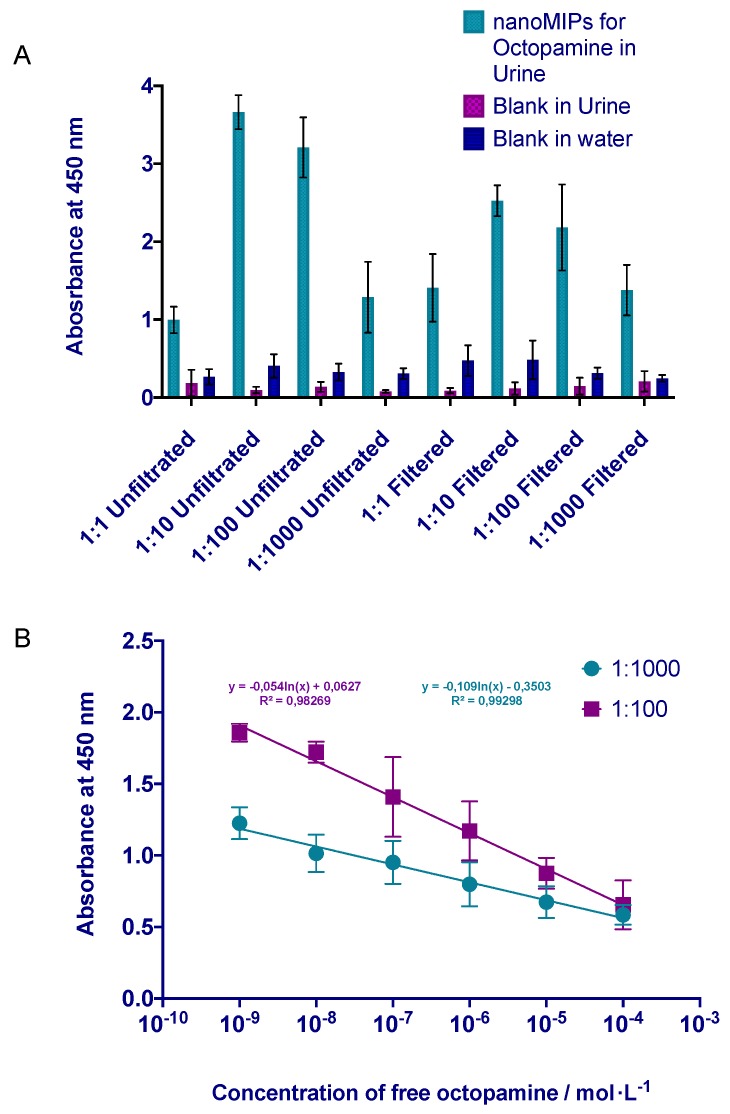
(**A**). Optimisation of the MINA assay in human urine. Urine was separated into two groups; filtrated and unfiltrated samples. Then the samples were separated into four different dilutions. (**B**) MINA calibration curve for the best two dilutions of filtrated human urine (1:100 and 1:1000). Error bars represent the standard deviation for experiments performed in triplicate.

After the experiments were performed in water, MINA was evaluated in the real human urine samples. Human urine is the most common matrix for analysis of forbidden substances in sport used for doping. Firstly, the assay had to be optimised in order to minimise interferences of the urine matrix and maximise the sensitivity of the method. Therefore, the absorbance of four different dilutions of the filtered and unfiltered samples was measured and compared in Figure 5A. Control experiments were performed in water and urine samples using uncoated microplates (blanks). In all the cases, the nanoMIPs assay prepared for octopamine revealed high selectivity compared with both types of blank experiments. Slightly lower absorbance was observed for the samples without dilutions and filtration, but already 1:10 dilutions significantly improved the signal. Based on the intensity of absorbance and lower interferences from the matrix, the best solution was chosen at 1:100 and 1:1000 dilution of the urine. Finally, the calibration curve for the detection of octopamine in urine samples was performed using two selected dilutions (see Figure 5B. The linearity of the assay was defined as r^2^ = 0.9827 and r^2^ = 0.9930 for the dilutions 1:100 and 1:1000, respectively. The assay presented a linear response in a concentration range of 1 nmol·L^−1^–0.0001 mol·L^−1^. The results indicated that free octopamine could be detected within five orders of magnitude of the concentration range plotted on a logarithmic scale. The LOD was 0.059 ± 0.00281 µg·mL^−1^ and the LOQ was 0.1947 ± 0.00915 µg·mL^−1^, it was calculated from 24 times the value of the standard deviation of the control (in the absence of octopamine).

**Figure 6:**
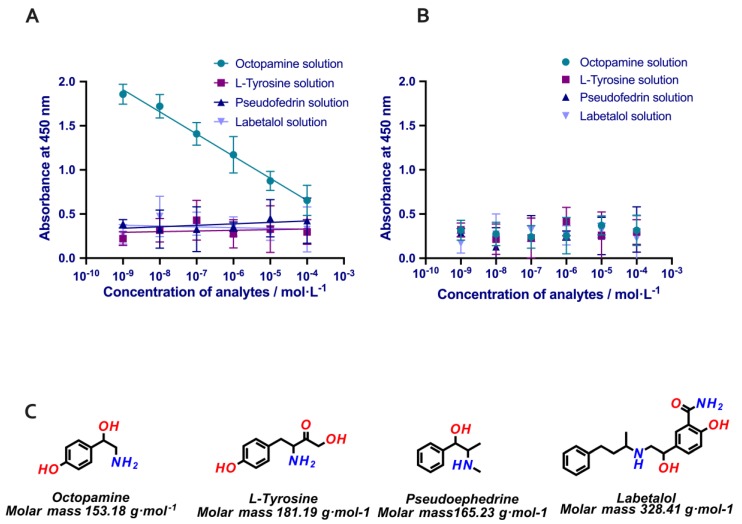
(**A**) Calibration curve for MINA performed in microplates with immobilised nanoMIPs for octopamine. Competitive assay performed with different concentrations of; octopamine, labetalol, pseudoephedrine and l-Tyrosine solutions in human urine samples. Error bars represent the standard deviation for experiments performed in triplicate. (**B**) MINA assay with nanoNIPs immobilised made for Atenolol. (**C**) Chemical structures of four drugs used in the cross reactivity assay.

Therefore, nanoMIPs proved their binding ability to octopamine, and the MINA assay affirmed good limits of detection in both sets of samples, water and urine. The next step was to evaluate the cross-reactivity of the assay in urine samples (see Figure 6). The experiments were done using three analytes related to octopamine. The first molecule was labetalol, which is also a banned molecule in sport. The other two selected molecules were analogues of octopamine (l-Tyrosine, the precursor of the biosynthesis of the octopamine and pseudoephedrine, molecule with a very similar molecular weight (the difference is less than 12 g·mol^−1^). The results of the cross reactivity clearly indicated significantly lower binding of the nanoMIPs to the analogue analytes, which competed very poorly with the octopamine conjugate. 

The results, along with previous experiments of the competitive MINA assay for the nanoMIPs and nanoNIPs (see Figure 3 and Figure 4) demonstrated a high specific affinity of the nanoMIPs for octopamine.

Additional experiments were performed to study the stability of the nanoMIPs coated in the ELISA microplate. In order to do that, the calibration curve was performed in the same conditions and using the same ELISA-coated microplate after one month of storage at the ambient temperature. It was found that the plate did not lose the original activity and the selectivity for octopamine. The results showed the plates coated with nanoMIPs could still recognise octopamine in the concentration range of 1 nmol·L^−1^ to 0.0001 mol·L^−1^ without changes in the limit of detection.

Finally, to demonstrate the applicability of the MINA assay, drinkable water and human urine were spiked with octopamine at the clinically relevant concentrations (see Table 2) between 0.05 and 50 µg·mL^−1^.

## 4. Conclusions

According to our knowledge, it is the first time when a molecularly imprinted nanoparticles assay (MINA) was investigated for detecting and quantifying substances banned by anti-doping regulation and organisation. In this work, we present the new way to detect octopamine in human urine and drinkable water samples, similar to conventional ELISA, but replacing traditional antibodies with nanoMIPs. The study confirmed that nanoMIPs could be used in the pseudo-ELISA with high specificity and sensibility for the detection of octopamine.

The nanoMIPs were characterised by using DLS and TEM, proving the nanosize and spherical shape of the nanoparticles with a homogeneous distribution of 137 ± 2.210 nm and low polydispersity index of 0.3205 ± 0.0121.

The MINA assay was able to detect octopamine in water within the range of 1 nmol·L^−1^–0.1 mol·L^−1^ with a detection limit of 0.047 ± 0.00231 µg·mL^−1^. In human urine samples, it was detected within the range of 1 nmol·L^−1^–0.0001 mol·L^−1^ with a detection limit of 0.059 ± 0.00281 µg·mL^–1^. In both cases, the limits of detection are at least 1 order of magnitude more sensitive than the HPLC MS/MS assay, and at least 2 orders of magnitude more sensitive than the traditional monoclonal antibody ELISA. We can conclude that MINA assay can be used to determine octopamine in water and human urine samples in the clinically relevant concentrations with a mean of the accuracy of 96–106%. Furthermore, the assay demonstrated high selectivity for octopamine with a low cross-reactivity. Therefore, MINA demonstrated performance comparable to traditional monoclonal antibodies and might open new possibilities of tracking prohibited substances in complex biological samples. It could also significantly improve the anti-doping system, providing a rapid and cost-effective alternative to the existing routine analytical methods for doping control. Additionally, the simplicity of the method of synthesis of nanoMIPs and analysis of pseudo-ELISA suggest that the MINA assay can be done for several analytes in a relatively short time.

## Figures and Tables

**Table 1:** Standard procedure for competitive MINA.

Procedure	Required Solution
1. NanoMIPs immobilisation	45 μL of nanoMIPs 0.056 mg·mL^−1^ (24 h ambient temperature, dark).
2. Washing	0.01 mol·L^−1^ of PBS (2 × 250 μL) pH 7.2.
3. Blocking agent	0.1% of BSA and 1% of Tween 20 in 0.01 mol·L^−1^ (300 μL, 2 h).
4. Washing	0.01 mol·L^−1^ of PBS (3 × 250 μL) pH 7.2.
5. Target and conjugate	100 μL of HRP-O conjugate (1:1200) and the standard solution of free octopamine 1 h.
6. Washing	0.1% of BSA and 1% of Tween 20 in 0.01 mol·L^−1^ (3 × 300 μL).
7. Substrate addition	100 μL of TMB solution, 10 min.
8. Stopping solution	100 μL of 0.5 mol·L^−1^ H_2_SO_4_.

**Table 2:** Recovery analysis of drinkable water and human urine samples spiked with octopamine.

Sample	Spiked (μg·mL^−1^)	Found (μg·mL^−1^)	Recovery (%)
Drinkable water 1	0.08	0.0851	106.4
Human urine 1	0.08	0.0774	96.8
Drinkable water 2	0.5	0.4810	96.2
Human urine 2	0.5	0.515	103
Drinkable water 3	50	51.6	103.2
Human urine 3	50	49.5	99

## Supplementary Materials

Details about size distribution on DLS and optimisation of the concentration of nanoMIPs in MINA are available online at https://www.mdpi.com/2073-4360/11/9/1497/s1.
