# Supplementary material for: Molecularly Imprinted Nanoparticles Assay (MINA) in Pseudo ELISA: An Alternative to Detect and Quantify Octopamine in Water and Human Urine Samples
Source: Polymers (Basel). 2019 Sep 13;11(9):1497. doi: 10.3390/polym11091497 (PMC6780943; doi:10.3390/polym11091497)
Supplement: Supplementary file 1 [file polymers-11-01497-s001.pdf]

Molecularly imprinted nanoparticles assay (MINA) in pseudo ELISA: An alternative to detect and quantify Octopamine in water and human urine samples

Ewa Moczko[a], Richard Díaz[b], Bernabé Rivas[b], Camilo García[c], Sergey Piletsky[d], Eduardo Pereira[e] and César Cáceres[b]*

[a.] *Departamento de Química Ambiental, Facultad de Ciencias, Universidad Católica de la Santísima Concepción, Concepción, Chile.*

[b.] *Departamento de Polímeros, Facultad de Ciencias Químicas, Universidad de Concepción, Chile.*

[c.] *Departamento de Ciencias Biológicas y Químicas, Facultad de Recursos Naturales, Universidad Católica de Temuco, Temuco, Chile.*

[d.] *Chemistry Department, College of Science and Engineering, University of Leicester, UK.*

[e.] *Departamento de Química Analítica e Inorgánica, Facultad de Ciencias Químicas, Universidad de Concepción, Chile.*

*Author to whom correspondence should be addressed: E-mail: cecaceres@udec.cl, T: +560412204171

Supplementary tables and figures

Table S1. DLS diameters of 10 different samples of nanoMIPs made for octopamine collected at 60 ºC.

G(d)	d (nm)	d (nm)	d (nm)	d (nm)	d (nm)	d (nm)	d (nm)	d (nm)	d (nm)	d (nm)
25,85	5,67E+01	5,69E+01	5,59E+01	5,72E+01	5,81E+01	5,91E+01	6,03E+01	5,88E+01	5,71E+01	5,94E+01
43,97	6,87E+01	6,91E+01	6,81E+01	6,93E+01	7,04E+01	7,15E+01	7,28E+01	7,10E+01	6,92E+01	7,16E+01
58,47	7,82E+01	7,89E+01	7,77E+01	7,90E+01	8,03E+01	8,14E+01	8,27E+01	8,07E+01	7,88E+01	8,12E+01
70,15	8,67E+01	8,75E+01	8,63E+01	8,75E+01	8,90E+01	9,02E+01	9,14E+01	8,92E+01	8,73E+01	8,96E+01
79,68	9,47E+01	9,58E+01	9,45E+01	9,57E+01	9,74E+01	9,85E+01	9,97E+01	9,74E+01	9,54E+01	9,77E+01
87,17	1,03E+02	1,04E+02	1,02E+02	1,04E+02	1,05E+02	1,07E+02	1,08E+02	1,05E+02	1,03E+02	1,05E+02
92,86	1,10E+02	1,12E+02	1,10E+02	1,12E+02	1,14E+02	1,15E+02	1,16E+02	1,13E+02	1,11E+02	1,13E+02
96,85	1,18E+02	1,20E+02	1,19E+02	1,20E+02	1,22E+02	1,23E+02	1,24E+02	1,21E+02	1,19E+02	1,21E+02
99,21	1,27E+02	1,28E+02	1,27E+02	1,28E+02	1,30E+02	1,31E+02	1,32E+02	1,29E+02	1,27E+02	1,29E+02
100,00	1,35E+02	1,37E+02	1,36E+02	1,37E+02	1,39E+02	1,40E+02	1,41E+02	1,38E+02	1,36E+02	1,38E+02
99,21	1,45E+02	1,47E+02	1,46E+02	1,46E+02	1,49E+02	1,50E+02	1,51E+02	1,48E+02	1,46E+02	1,47E+02
96,85	1,55E+02	1,57E+02	1,56E+02	1,56E+02	1,60E+02	1,60E+02	1,61E+02	1,58E+02	1,56E+02	1,57E+02
92,86	1,66E+02	1,69E+02	1,67E+02	1,68E+02	1,71E+02	1,72E+02	1,72E+02	1,69E+02	1,67E+02	1,68E+02
87,17	1,78E+02	1,82E+02	1,81E+02	1,81E+02	1,84E+02	1,85E+02	1,85E+02	1,81E+02	1,80E+02	1,80E+02
79,68	1,93E+02	1,97E+02	1,96E+02	1,96E+02	2,00E+02	2,00E+02	2,00E+02	1,96E+02	1,94E+02	1,95E+02
70,15	2,11E+02	2,16E+02	2,14E+02	2,14E+02	2,18E+02	2,19E+02	2,18E+02	2,14E+02	2,12E+02	2,12E+02
58,47	2,34E+02	2,40E+02	2,38E+02	2,37E+02	2,42E+02	2,42E+02	2,41E+02	2,37E+02	2,35E+02	2,34E+02
43,97	2,66E+02	2,73E+02	2,72E+02	2,70E+02	2,76E+02	2,76E+02	2,74E+02	2,69E+02	2,68E+02	2,66E+02
25,85	3,23E+02	3,32E+02	3,31E+02	3,27E+02	3,35E+02	3,34E+02	3,31E+02	3,25E+02	3,25E+02	3,20E+02

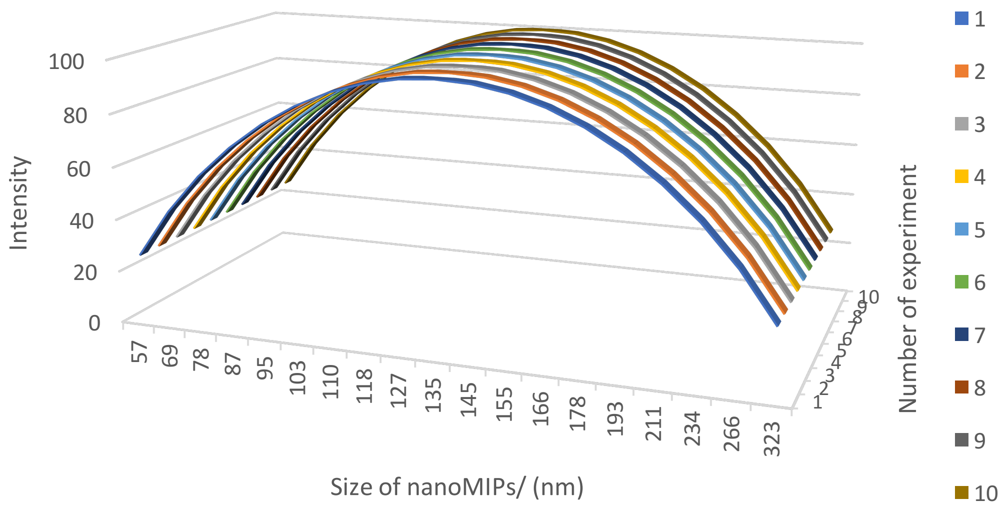

Supplementary Fig. S1. Diagram of the size distribution of nanoMIPs eluted at 60 ºC, plot in logarithmic scale.

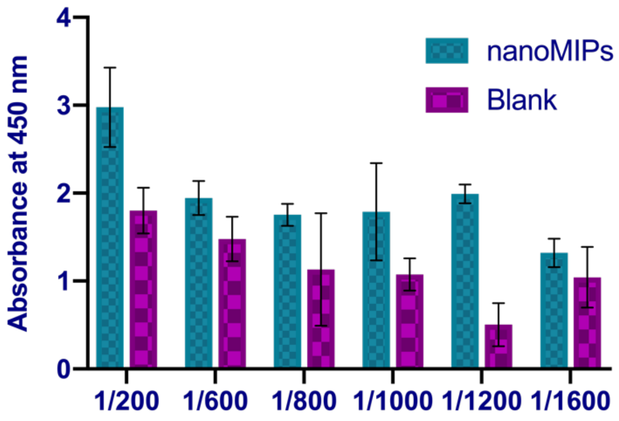

Supplementary Fig. S2. Optimization of the concentration of HRP-O using 6 dilutions of unstock HRP-O and the microplates with the immobilised nanoMIP for octopamine and uncoated microplates (Blank). Error bars represent the standard deviation for experiments performed in triplicate.

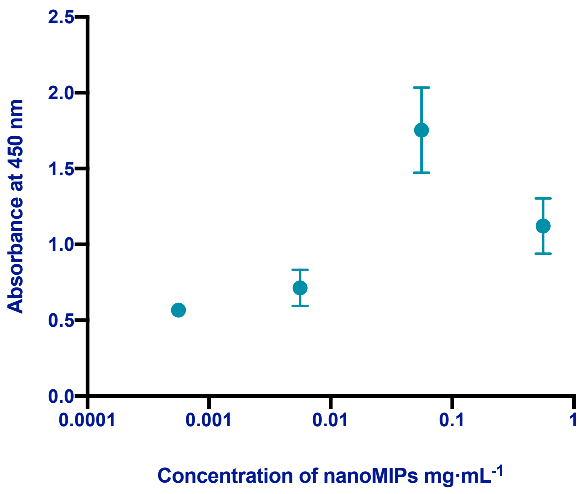

Supplementary Fig. S3. Optimisation of nanoMIPs concentration, each microplate was coated with different concentrations of nanoMIPs from 0.00056 to 0.56 mg·mL^{-1}. The HRP-O conjugate at 1:1200.

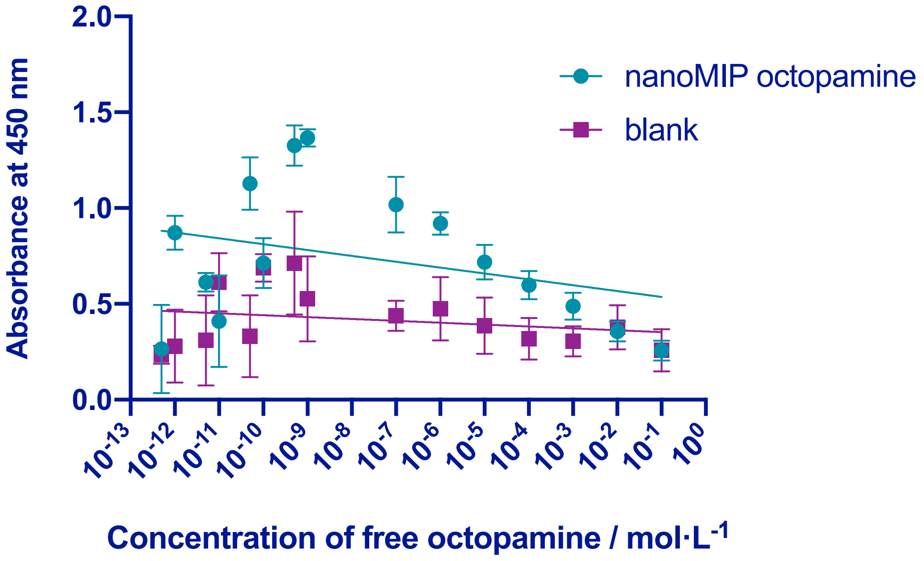

Supplementary Fig. S4. Optimization of the linear range of work for MINA competitive assay. Light blue line indicates binding of octopamine to octopamine specific nanoMIPs, (squares). Purple line indicates binding of octopamine to blank, uncoated microplates. Error bars represent the standard deviation for experiments performed in triplicate.
